# A novel deforestation risk and baseline allocation model for the next generation of nested REDD+ projects

**DOI:** 10.1038/s41598-024-65141-x

**Published:** 2024-07-02

**Authors:** Jeremy Freund, Maren Pauly, Will Gochberg, Emily M. Dangremond, Mike Korchinsky

**Affiliations:** 1 Wildlife Works, Mill Valley, CA USA; 2 Department of Research and Evaluation, Everland, New York City, NY USA

**Keywords:** Environmental impact, Climate-change mitigation

## Abstract

Nature-based solutions that use a counterfactual scenario depend heavily on the methodology used to determine the business as usual (BAU) case, i.e., the “baseline.” Reducing emissions from deforestation and forest degradation (REDD+) projects traditionally set baselines using a “reference area” as a control for estimating BAU deforestation and emissions in the treatment (project) area. While the REDD+ market is shifting from project-based to nested approaches as countries increase their efforts to meet nationally determined contributions (NDCs) to the Paris agreement’s global climate target, methodologies for allocating national baselines are not yet formalized and tested, despite an urgent need to scale the market. We present a novel method for mapping deforestation risk and allocating national forest reference emission levels (FREL) to projects: baseline allocation for assessed risk (BAAR). This approach provides a spatial predictor of future deforestation using a dynamic vector, and a method for allocating a FREL to differentiated risk areas at the project level. Here, we present BAAR using 34 REDD+ projects in the Democratic Republic of the Congo (DRC). We demonstrate the importance of risk-based FREL allocations to balance fitness for purpose and scientific rigor. We show how BAAR can be used by governments to focus voluntary carbon market finance in areas at highest risk of imminent deforestation, while maintaining alignment with nationally determined contribution (NDC) goals.

## Introduction

As the world grapples with climate change scenarios in which keeping warming below 2 ℃ is less and less likely^1^, natural climate solutions are being heralded as powerful mitigation strategies. Reducing deforestation—particularly in the tropics^2^—is one of the most immediate, effective natural climate solutions available for mitigating greenhouse gas emissions at scale^2,3^, as it preserves existing mature trees rather than waiting for newly planted trees to grow to maturity. When implemented under a market-incentivized reducing emissions from deforestation and forest degradation (REDD+) project, the drivers of deforestation are addressed through a systematic approach that results in impactful benefits for local communities and wildlife^4^. REDD+ is a mechanism to slow deforestation through performance payments for emissions reductions to developing countries, first developed in 2005 through the United Nations Framework Convention on Climate Change (UNFCCC)^5^. Emissions reductions that are financed through international carbon markets have enormous potential to contribute to achieving global climate goals, especially compared to a scenario in which countries attempt to meet their respective goals independently^6^.

For activities that reduce emissions, emissions reductions are calculated by comparing project area GHG emissions to a counterfactual scenario, referred to as a baseline. In the voluntary carbon market (VCM), verification of these emissions reductions generally happens when a third-party organization conducts an audit of a REDD+ project against a standard (e.g., the Verified Carbon Standard or Plan Vivo). After the project is assessed by a third party validation and verification body (VVB), credits are issued based on tonnes of CO_2_ equivalent GHG emissions avoided by the REDD+ project compared to the baseline scenario—known as verified emissions reductions (VERs).

While similar mechanisms to reduce deforestation have been randomly assigned to facilitate causal identification^7^, in practice REDD+ projects are not developed in randomly selected forest areas or communities, making it difficult to measure projects’ causal effect. In the absence of randomization, a variety of methods have been developed to establish plausible reference regions which serve as the basis of comparison to project areas. The underlying mechanics of baseline-setting methodologies can significantly affect the number of carbon credits issued, with subsequent revenue implications for local communities, governments, investors, and project developers^2^. Considering the climate finance invested in REDD+ projects and the application of VERs towards corporate claims, the endeavor has been under intense scrutiny. Recent academic studies have tested the application of statistical techniques (difference-in-differences and synthetic controls) to estimate the effect of REDD+ interventions on land use^8–11^. While evaluating the accuracy ex-post facto is important, recent attempts^11,12^ have been applied in such a way to elicit highly uncertain conclusions^13,14^. Irrespective of the baseline criticisms, the effectiveness of project-based REDD+ in reducing deforestation has been demonstrated in many geographies^15^. Based on the evidence of success thus far, REDD+ is ready to scale.

Several studies suggest that the efficacy of REDD+ as a market-based countermeasure to extraction relies on role-specific allocation of incentives^16–19^. To complement a legislation/enforcement approach typically employed at jurisdictional scales, local performance targets offer communities financial incentive for protecting their own forests. As such, REDD+ performance to date has been primarily project-based, but remains poorly connected to host country REDD+ strategies or NDC accounting.

When implemented at the national (or sub-national jurisdictional) level, nesting attempts to close this gap by integrating projects into a national REDD+ program. A nested baseline approach has two main steps: the creation of a jurisdictional forest reference level (FREL) and the allocation of the FREL to projects. The FREL, which is an estimate of business-as-usual emissions from deforestation jurisdiction-wide, can be calculated as a simple historical average or through more complex statistical modeling. This study addresses the spatial allocation of a FREL, as opposed to its initial calculation. There are multiple ways to allocate FRELs to projects: we present one such method here.

It is worth noting that the distinction between jurisdictional FREL-based nested baselines and project-scale baselines is fundamentally a matter of applicability rather than superiority. The nested baseline approach prioritizes consistency across jurisdictions, employing an inherently conservative methodology to ensure broad risk distribution and alignment with jurisdictional climate change objectives. This approach is increasingly recognized in academic and policy circles as the pathway forward that best promotes international consistency and collaboration towards global climate goals. In contrast, project-scale baselines provide localized precision, adept at capturing nuanced deforestation dynamics within specific areas. This study focuses on the nested approach, not to diminish or compete with the value of project-specific baseline setting methodologies, but to align with the evolving market consensus that views the jurisdictional/nested framework as a cornerstone for achieving standardized, transparent, and scalable impacts in forest conservation and climate mitigation.

Beyond consistency, national nesting systems offer several additional benefits: firstly, by clarifying the mechanisms needed to incentivize VCM stakeholders and investors for project-scale activities, nesting allows REDD+ to be financed and implemented site-by-site on private, public and community lands. Crucially, nesting also enables the establishment of performance targets tailored to local conditions. This approach incentivizes communities to garner finance for successfully reducing deforestation in the forests they manage, regardless of the national program’s overall success in lowering emissions—a scale at which local communities have minimal influence.

In turn, this allows governments to direct financial resources strategically towards fulfilling their national development goals. National nesting establishes a transparent framework for regulating and distributing climate finance across multiple scales. This level of clarity is crucial for effective governance and enforcement. For example, national governments hold the authority to enforce timber harvesting laws, resolve legal disputes, and regulate land use—factors that can significantly enhance the effectiveness of REDD+ activities^20^. Nesting can also mitigate ‘leakage,’ where deforestation simply shifts locations rather than decreasing due to REDD+ efforts^21^. Importantly, national risk mapping allows for equitable baseline allocation across the country.

**Figure 1:**
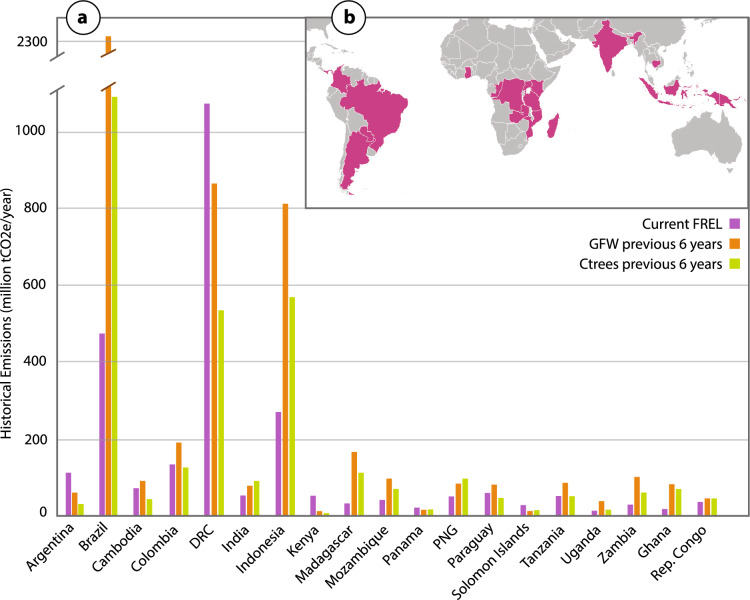
(**a**) Current country-level forest reference emissions levels (FRELs) compared to historic (2015–2021) global forest watch (GFW; Hansen et al. 2013) and Ctrees datasets for a subset of countries (**b**) housing REDD+ projects. The historic reference periods underpinning country FRELs vary: Argentina: 2002–2013, Brazil: 2016–2021, Cambodia: 2011–2018, Colombia: 2008–2017, DRC: 2000–2014, India: 2000–2008, Indonesia: 2006–2020, Kenya: 2002–2018, Madagascar: 2005–2015, Mozambique: 2003–2013, Panama: 2006–2015, PNG: 2001–2013, Paraguay: 2012–2019, Solomon Islands: 2001–2017, Tanzania: 2002–2013 (mainland), Uganda: 2000–2015, Zambia: 2009–2018, Ghana: 2001–2015, Republic of the Congo: 2000–2012. Map developed in Adobe Illustrator 26.2.1 (https://www.adobe.com/products/illustrator.html) using Robinson projection global basemap (https://en.m.wikipedia.org/wiki/File:BlankMap-World.svg).

Finally, nesting has been identified by an increasing number of REDD+ countries as an effective mechanism for achieving their national climate ambitions. Allocation of the national FREL to projects (Fig. 1) aligns the VCM with governments’ nationally determined contributions (NDCs) under the UNFCCC’s Paris agreement^22^, bolstering the global response to climate change.

**Figure 2:**
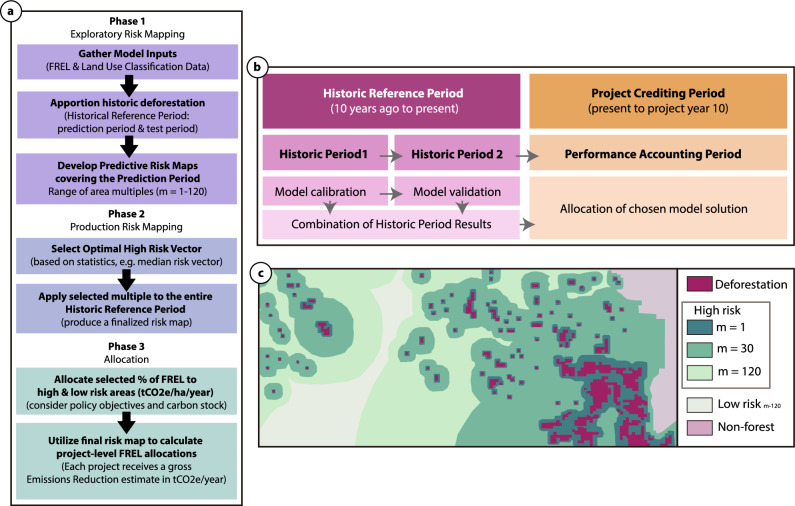
(**a**) The three phases of the approach for risk-based reduction of emissions (BAAR); (**b**) the temporal splitting of the historic reference and project crediting periods required for deforestation risk model calibration, validation and eventual allocation; (**c**) an example of predicted high risk for various vector-based multiples (m = 1, 30, 120) and low risk deforestation pixels for m = 120; produced for the model validation period based on the model calibration period.

Here we introduce a new deforestation risk mapping and allocation benchmarking approach for application to nested REDD+ : the baseline allocation for assessed risk (BAAR; Fig. 2). In this approach, the most significant spatial driver of future deforestation is past deforestation^23^, applied to remaining forest cover using a dynamic, vector-based (non-uniform) approach. BAAR allows for easy calculation of site-scale baselines that allow for the distribution of incentive to areas determined to be at high risk of near-term deforestation. Crucially, BAAR balances the fitness for purpose required for an incentive mechanism intended for rapid growth, with the high scientific integrity required by claimants in the VCM.

We have undertaken exploratory testing of BAAR in a series of tropical forest countries, resulting in predictive risk maps and allocated reference levels. The DRC’s large tropical forest estate, high forest carbon stocks and increasing deforestation rates yield a high national FREL (Fig. 1a). REDD+ is a compelling mechanism for avoiding deforestation and degradation of DRC’s carbon rich forest, and presents an enormous opportunity for climate change mitigation and forest community self-empowerment. In this study we present initial results for several avoidance projects in the DRC.

We present the BAAR model as a tool meant for practical use in the REDD+ sector of the carbon market, where host country governments are quickly gaining interest in crafting regulation to achieve a number of policy goals. Therefore, we are explicit about the tradeoffs inherent in the choice of a risk map, the proportion of reference level allocated to high-risk forests, and the ability to achieve national GHG emission reductions targets. We also observe that the relationship among these variables cannot be explained by a universal, consistent pattern, even among projects within the same jurisdiction. Quantifying these tradeoffs is an important step towards practical application of BAAR in the REDD+ sector. For example, there may be a set of jurisdictional risk maps that meet some threshold of acceptable accuracy, which allows for the choice among this set of the map that most effectively incentivizes nested projects to operate in areas of high risk of deforestation, or alternatively the map that most closely aligns with host country climate targets. The credibility of emission reductions calculated using BAAR baselines is preserved nonetheless. Nested projects may rely on monitoring, reporting, and verification (MRV) conducted at the jurisdictional level, and the allocation of a FREL creates a cap on total ERs available for projects.

## Methods

BAAR integrates three phases (Fig. 2a): (1) exploratory risk mapping, (2) production risk mapping, and (3) allocation. Below, we detail this approach using the DRC as an example.

### Phase 1: exploratory risk mapping

#### Phase 1a: gather model inputs

The BAAR risk map algorithm requires a series of land classification maps covering a historic reference period of 10+ years as its initial input—those submitted as the basis of developing a national or jurisdictional forest reference emission level (FREL) accepted by an international body of experts^22,24^. A FREL is expressed as the tonnes of CO_2_ equivalent (tCO_2_e) per year measured over a historic (reference) period and projected into the future for a defined validity period. This annual tCO_2_e value represents the value by which any forest removals or emissions are compared against for the purpose of implementing REDD+ activities. In order to allocate this FREL (Phase 2), a detailed map of future deforestation risk is required (Phase 1; Fig. 2). For the DRC, the historic reference period inputs for the risk map utilize the existing national FREL^24^ accepted by the UNFCCC secretariat^25^, which includes activity and forest benchmark data (Supplementary Fig. 1).

The activity data and forest benchmark maps (Supplementary Fig. 1) underlying the DRC FREL were used as the model inputs, covering 14 years (2000–2014) at a spatial resolution of 30 m. Following initial scanning of the 2000 forest cover map, areas with < 5000 ha of contiguous forest were removed from further analysis—leaving a total of 140,861,872 ha of intact forest cover for risk mapping. The FREL’s historic period was split into two time spans to be utilized as the prediction period and the test period (Fig. 2b). The total historic period covered 2000–2014, split into the model calibration years (2000–2010) and model validation years (2010–2014) as a basis for predicting a project crediting period from 2015–2019.

#### Phase 1b: apportion historic deforestation

The level of deforestation was predicted spatially in the BAAR by applying varying multiples of contiguous patches of historical deforestation greater than 0.18 ha (two image pixels) in size, as historic deforestation is evidenced to be the most important variable impacting future deforestation risk^26,27^. This was undertaken using ArcGIS by setting a buffer of predicted deforestation at various dynamic distances from each existing deforestation patch using a series of vector-based multiples (“m” values) using the ArcGIS buffer function. For example, an m-value of 60 represents a risk buffer set at 60 times the area of each local historic deforestation patch Eq. (1). This method assumes that risk of future deforestation is highest near previous deforestation^28^ and that larger historic deforestation patches represent greater risk of future deforestation adequately capturing deforestation frontiers^29^.

1 $${r}_{high}^{[n]}=\sqrt{\frac{\left({a}_{p}^{[n]}\cdot m\right)+ {a}_{p}^{[n]}}{\pi }} - \sqrt{\frac{{a}_{p}^{[n]}}{\pi }}$$

where;

${r}_{high}^{[n]}$ = the radius of the high risk buffer created for each local deforestation patch.

${a}_{p}^{[n]}$= The area of each local deforestation patch.

$m$ = The area multiple for the high risk polygon to the local deforestation patch.

$n$ = number of contiguous local deforestation patches observed within the historical reference period.

An exploratory analysis was undertaken for the DRC using a series of different m values. A total of 14 multiples (m = 1, 2, 3, 6, 9, 12, 15, 30, 45, 60, 75, 90, 105, 120) were tested to develop a series of predictive maps. Auxiliary risk variables (e.g. distance to rivers, roads) were tested to determine their predictive power. In cases where they did not improve the predictive power of the model, the variables were excluded from the analysis. A series of risk maps were developed covering the historic period and all risk multiples, with the total mapped area (excluding the forest patches that do not pass the threshold) set to two risk strata: high-risk and low-risk.

### Phase 2: production risk mapping

To calculate the risk map’s predictive ability, the percentage of deforestation that actually occurred in the test (model validation) period was compared to the area mapped as “high-risk” according to the series of risk maps produced in Phase 1. This provides an estimation of the predictive power (PP) of the model Eq. (2)—ranging from 0–100%—with higher percentages demonstrating higher proportions of the actual deforestation captured within the high-risk prediction area.

2 $$PP= \frac{{A}_{def high}}{{A}_{def}}$$

where;

${A}_{def high}$ = The area of deforestation captured within the high-risk predictive area for the validation period.

${A}_{def}$= Total area of deforestation for the validation period.

It is recommended to maintain the calibration and validation periods as identical. However, in most cases, national FRELs are not calculated over uniform time periods. If the calibration and validation periods are not identical, predictive power should be scaled according to their difference. The scaled predictive power (PPs) is calculated following Eq. (3):

3 $$PPs = PP \times \left(\frac{{n}_{years \;calibration}}{{n}_{years \;validation}}\right)$$

The risk map precision was calculated using Eq. (4). Higher precision indicates the capture of higher proportions of the actual deforestation in a smaller high-risk prediction area.

4 $$Pr= \frac{{A}_{def}}{{A}_{high}}$$

where;

${A}_{def}$= Total deforestation area for the validation period.

${A}_{high}$= Area of the high-risk predictive area.

**Figure 3:**
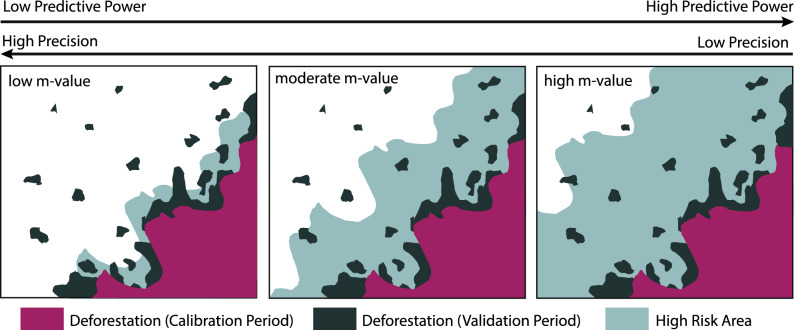
The relationship between m-values, precision and predictive power of the BAAR model.

Beyond PP and Pr, it is challenging to quantify the error of a forest loss risk map as its intent is to highlight likelihood of forest loss rather than forecast specific instances of loss. PP can be misleading, as higher values (i.e. predictive power close to 100%) do not necessarily reflect a more realistic map. For example, higher m-value maps produce larger predictive areas of risk—thus, a higher proportion of the actual deforestation is captured within the high-risk area (Fig. 3). However, due to the extensive areas designated as high-risk, the precision is relatively low. This is because a significant portion of these high-risk areas do not experience deforestation during the validation period, as indicated by low Pr values. The opposite is true for the low m-value maps—precision may be relatively high, but the proportion of actual deforestation that occurred outside of the high risk area increases. Considering these challenges, relying solely on PP and Pr as metrics to select the optimal m-value map for FREL allocation may not provide the most comprehensive assessment.

The first step in allocating the FREL tCO_2_e to the risk maps is to select the most appropriate m-value map set that balances: (1) scientific rigor (realistic uncertainty that can be attained in the landscape), (2) realistic conservation goals (minimum achievable effectiveness in protecting the forest and reducing local emissions), and (3) incentive for investment (adequate reference levels to meet the country’s NDC goals).

A proportion of the FREL must be allocated towards high-risk and low-risk areas, selected based on the local policy objectives that incentivize conservation. The country may wish to allocate a majority share of the FREL to the high risk areas, in which case they would incentivize the protection of the forests with the greatest probability of near-term deforestation. They may instead allocate a larger proportion of the FREL to the low-risk areas if they wish to incentivize communities whose land has been historically well-protected, but requires new or continuing finance.

While BAAR is meant to be customized by national governments to assess and manage risks, here we provide an example to demonstrate how BAAR can be used to optimally allocate resources based on the required effectiveness of different projects to mitigate those risks.

Minimum project efficacy (MPE) is the percentage of this high-risk area that must remain forest to meet a country’s stated NDC goal, assuming that all remaining forests are part of conservation projects like REDD+ . The MPE was calculated for each area multiple using Eq. (5). Some Parties communicate their NDC target in tCO_2_e, others in percentage form. We convert the former into percentages before inputting them into the BAAR model, and use AFOLU-specific targets where they exist.

5 $$MPE = \frac{NDC}{PP}$$

where;

$NDC$ = The current nationally determined contribution commitment made by a country to reduce greenhouse gas emissions and adapt to the impacts of climate change as part of the Paris agreement, expressed as a percentage.

$$PP$$ = Predictive power.

Note that the denominator of MPE ranges 0–1, increasing with a risk map’s predictive power. A perfect map results in an MPE that equals the NDC target: in that case, for example, projects in a host country with an NDC target of 30% would need to reduce emissions by 30% compared to a business as usual scenario to align with the NDC. For maps with weaker predictive power, projects would need to reduce emissions by more than 30% to align with the same NDC target. The required MPE is an important measure to consider when allocating the FREL using the selected risk map. Based on the calculated PP and MPE for different area multiples, we selected an example map choice for further analysis. This ensures that allocation using the selected risk map can achieve the NDC goal at an achievable project efficacy, whilst maintaining a reasonable model predictive power. The selected risk map was then calculated using deforestation data across the entire historic period (calibration and validation periods).

### Phase 3: allocation

The allocation of the FREL to high and low risk areas of the selected risk map was weighted based on the relative carbon stock per area according to the main forest types in the DRC (Table 1; Supplementary Fig. 1).

**Table 1:** The main forest types and relevant carbon stocks in the DRC (MECNT, 2018); total area represents total cover of each type in the country.

Official forest classification	Translation	Mean carbon stock (tCO_2_e/ha)	Total area (ha)
Forêt dense humide sur terre ferme (FDHTF)	Dense humid terrestrial forest	747	98,092,159
Forêt dense humide sur sol hydromorphe (FDHSH)	Dense humid forest terrestrial forest on hydromorphic soil	718	16,490,795
Forêt secondaire (FSc)	Secondary forest	409	16,188,932
Forêt sèche ou forêt claire (FSFC)	Dry or open forest	245	23,942,665
Savane	Savanna	87	68,686,034

Once the maps were finalized and weighted by carbon stock, the FREL was distributed directly to site-scale activities based on current projects and known potential future projects in the DRC (Supplementary Fig. 2). Each project receives a nested baseline (reference level) in tCO_2_e/year—allocated to the project accounting area (PAA)—a subset of the total project area (PA) that is 100% forest for 10 years prior to project initiation.

## Results

### Deforestation prediction outputs

A total of 14 multiples, represented with the variable *m*, spanning a range from 1–120 were used to develop a series of predictive maps for the DRC (Table 2), resulting in scaled predictive powers (PPs) ranging from 30.3–195%, precisions from 4.1–56.7% and required minimum project effectiveness (MPE) scores from 10.8–69.2%. The m value is the area-multiple of each predicted high-risk buffer polygon to the area of the historical deforestation patch it was based on. However, at scale, as the ‘area multiple’ column demonstrates, an m-value of 6, for example, does not actually equate to an aggregate area multiple of 6× of the aggregate area of historical deforestation; this is because as the individual polygons increase in size they overlap, reducing the total area predicted as high-risk.

**Table 2:** DRC predictive maps statistical outputs for vector-based multiples (m = 1–120) covering the historic period.

Map (m value)	Aggregate area multiple of predictive area to historic deforestation	Predictive power (PP) (%)	Scaled predictive power (PPs) (%)	Precision (Pr) (%)	Efficiency (%)	FREL proportion allocated to high risk (%)	Required minimum project efficacy (MPE) (%)
1	1.02	12.1	30.3	56.7	6.88	90.0	69.2
2	1.67	19.0	47.5	34.4	6.54	90.0	44.2
3	2.14	24.0	59.9	26.9	6.45	90.0	35.1
6	3.36	33.9	84.7	17.1	5.80	90.0	24.8
9	4.24	40.3	100.8	13.6	5.47	90.0	20.8
12	4.96	45.0	112.5	11.6	5.23	90.0	18.7
15	5.57	48.7	121.6	10.3	5.03	90.0	17.3
30	7.83	59.8	149.4	7.4	4.40	90.0	14.1
45	9.41	65.8	164.5	6.1	4.03	90.0	12.8
60	10.65	69.7	174.4	5.4	3.77	90.0	12.0
75	11.68	72.6	181.5	4.9	3.58	90.0	11.6
90	12.57	74.8	187.0	4.6	3.43	90.0	11.2
105	13.35	76.6	191.4	4.3	3.30	90.0	11.0
120	14.04	78.0	195.0	4.1	3.20	90.0	10.8

The proportional area predicted to be under high vs. low risk of deforestation was varied across the m-values tested (Table 3), with lower m-values yielding less high risk area due to the relative size of the risk vector.

**Table 3:** The total (and proportional) high and low risk areas predicted across the series of m-values tested in BAAR.

m	High risk area (ha)	High risk area (%)	Low risk area (ha)	Low risk area (%)
1	6,393,251	4.55	134,229,295	95.45
2	10,051,745	7.15	130,570,801	92.85
3	12,582,332	8.99	127,438,667	91.01
6	18,938,742	13.47	121,683,804	86.53
9	23,357,074	16.61	117,265,472	83.39
12	26,908,764	19.14	113,713,782	80.86
15	32,616,192	23.15	108,245,680	76.85
30	43,428,608	30.83	97,433,263	69.17
45	50,811,803	36.07	90,050,069	63.93
60	56,528,478	40.13	84,333,394	59.87
75	61,219,262	43.46	79,642,610	56.54
90	65,205,170	46.29	75,656,701	53.71
105	68,683,185	48.76	72,178,687	51.24
120	71,760,988	50.94	69,100,883	49.06

### DRC FREL allocation to selected projects

**Figure 4:**
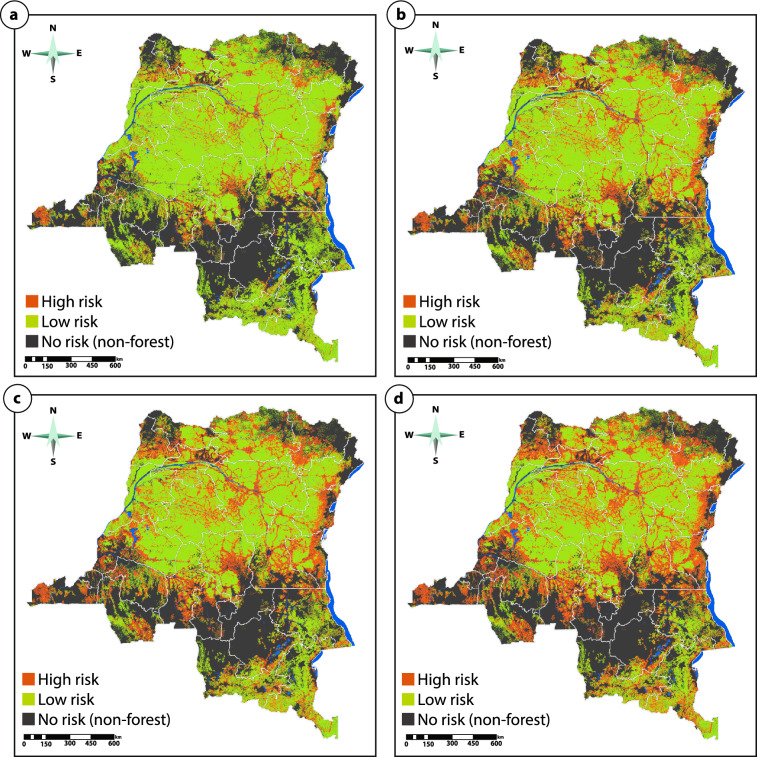
The BAAR deforestation risk maps for the DRC for (**a**) m = 15, (**b**) m = 45, (**c**) m = 75 and (**d**) m = 120 using the historic reference period of 2000–2014 (calibration: 2000–2010, validation: 2010–2014). Map developed using QGIS 3.28 (https://www.qgis.org/en/site/forusers/download.html) with post-processing in Adobe Illustrator 26.2.1 (https://www.adobe.com/products/illustrator.html).

Based on exploratory analysis of the set of risk maps, the m = 45 area multiple map was selected as an example optimal map choice for project baseline allocation, based on the MPE (= 12.8%) being the median of all relevant area multiple results (MPE < 100%). This ensures that the selected risk map could achieve the DRC’s NDC goal (21%) at a 12.8% minimum project efficacy (assuming 100% project coverage of remaining forest at the end of the historic period) whilst maintaining a 65.8% predictive power of the model. The final risk map was executed using the selected value across the entire historic period (Fig. 4). Based on this risk map, 50,811,803 ha of forest (36%) were classified as high risk and the remaining 90,050,069 ha (64%) were classified as low risk.

**Figure 5:**
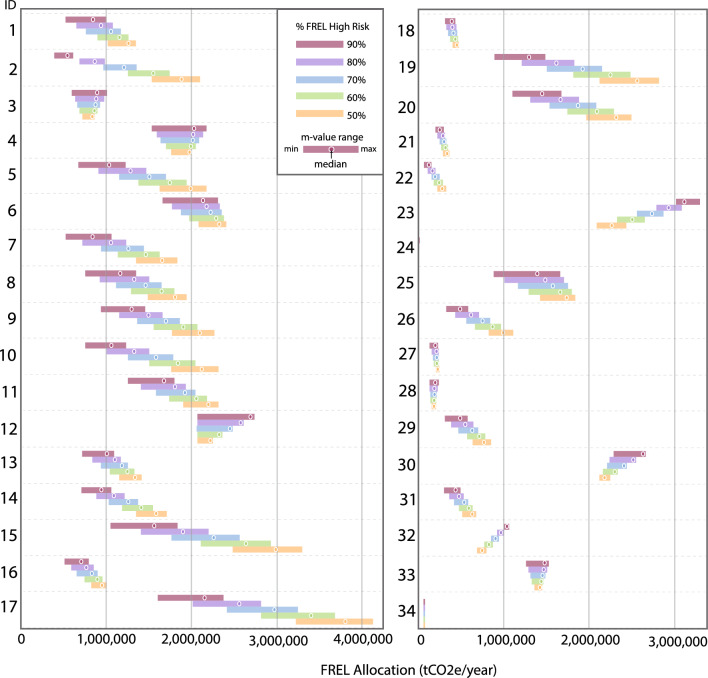
Potential project FREL allocation ranges based on BAAR for different m-values (m = 1–120) and a range of high:low allocation splits (90:10, 80:20, 70:30, 60:40, 50:50). See Supplementary Fig. 2 for project locations.

The average for the projected annual DRC FREL (1,078,235,018 tCO_2_e/year) can be allocated to the risk map using a range of high:low risk areas proportions (Fig. 4). The risk map and allocation of the FREL was applied to a series of REDD+ projects in various stages of development (Fig. 5; Supplementary Fig. 2, Supplementary Table 1) using a range of high:low risk ratios (from 90:10 to 50:50). On a project-by-project basis, the total results of all m-values and all FREL allocation ratios resulted in an average relative standard deviation (rSD) of ± 9.32% for the total tCO_2_e/year allocated. When the m-values were subsetted to a smaller range (m = 45–90), this allocation range was significantly reduced resulting in a rSD of ± 4.6%. In the majority of project cases (n = 26) the FREL allocated (tCO_2_e/year) decreases with the higher relative apportioning to high risk areas; a 90:10 risk split reduces FREL allocation to the project subset by an average of 27.0% compared to a 50:50 risk split.

**Figure 6:**
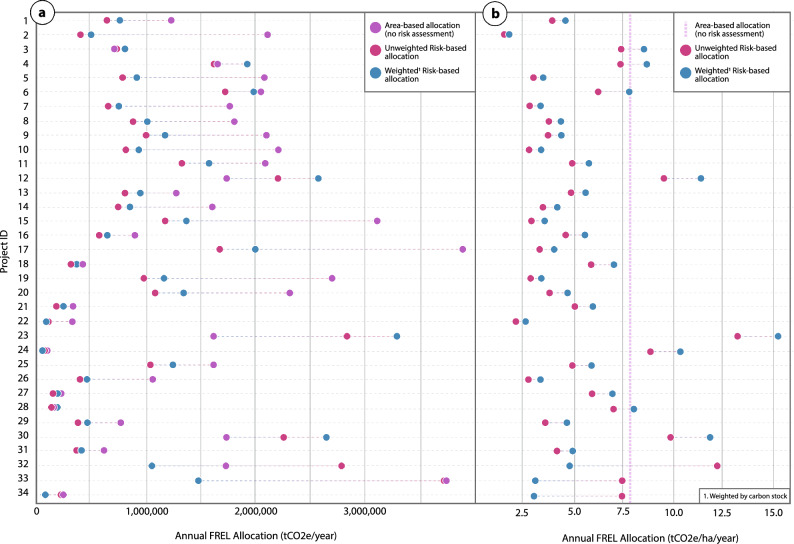
Annual FREL allocation (tCO_2_e/year) for a series of REDD + projects (Supplementary Table 1, Supplementary Fig. 2) in various stages of development calculated using the BAAR model (m = 45) for selected future and current REDD+ projects in the DRC based on (**a**) total project account areas (PAAs), and (**b**) normalized by PAA hectares. The model results are compared to a simple area-based FREL allocation with no relative deforestation risk calibration. BAAR FREL allocation results are demonstrated with and without carbon stock weighting.

Here we present the risk map with the median MPE selected from the set of maps with MPE < 100% (m = 45; Fig. 6). If the national DRC FREL (7.65 tCO_2_e/ha/year) was allocated simply based on the total accounting area of the project (without considering risk), the allocation per year would average 1,533,694 tCO_2_e/year for the above mentioned projects. BAAR initially results in a risk-based FREL allocation averaging 1,027,552 tCO_2_e/year (4.93 tCO_2_e/ha/year) for the selected projects, which resulted in an average of 1,050,528 tCO_2_e/year (5.84 tCO_2_e/ha/year) when weighted by carbon stock.

## Discussion

In this study, we have presented the BAAR method using 34 REDD+ projects in the Democratic Republic of the Congo. The exploratory risk mapping, production risk mapping, and allocation phases were described and demonstrated. A variable that should be considered when allocating a FREL is the minimum project efficacy (MPE); the MPE represents the total high-risk area that must be conserved (i.e. remain forest) to meet the country’s NDC goal. In this study, the m-value of 45, with a corresponding MPE = 12.8%, was chosen as an example outcome.

We have shown how a series of high:low ratio allocation splits result in a range of annual project-level FRELs (Fig. 5). In this run of the BAAR method, 90% of the FREL was allocated to high risk areas. The choices of which risk map to use and how to allocate a jurisdictional FREL are driven not only by concern for accurately monitoring and accounting for GHG reductions, but also by policy priorities, particularly when a national government has final say in these decisions. A government may wish to allocate a FREL that reflects average risk for areas at high risk of deforestation. At the same time, governments presumably wish to incentivize REDD+ activities in these high-risk areas by allocating more FREL where deforestation is likely. In such cases, a uniform FREL distribution, for example, would be inappropriate as it would greatly under-incentivize forest protection in some areas and over-incentivize it in others.

The allocation component of this model maintains the input historical emissions data (FREL) as is, instead differentiating the distribution of the FREL according to spatial risk patterns. As such, it can be applied to help governments align REDD+ baseline allocation and GHG accounting with their nationally determined contribution (NDC) toward the Paris agreement’s global temperature goal. This stands in contrast with other jurisdictional approaches that calculate or otherwise alter FREL components prior to their distribution.

**Figure 7:**
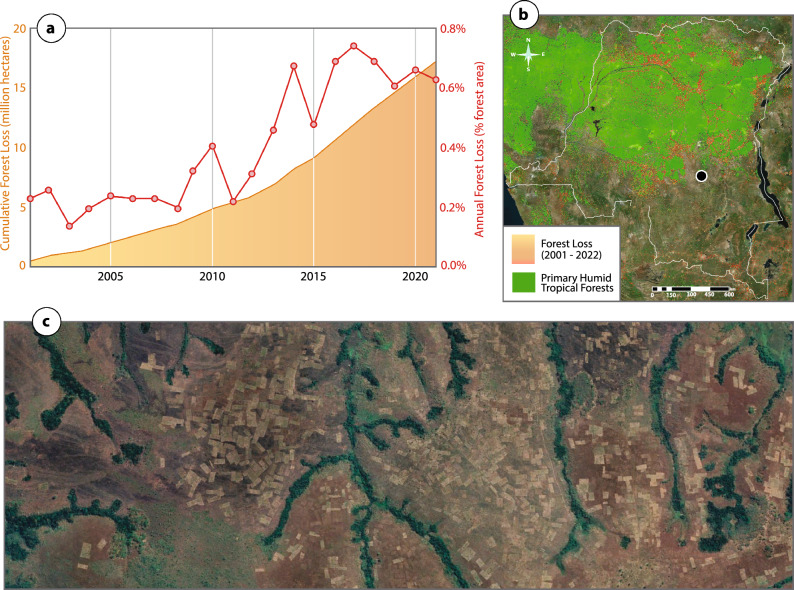
(**a**) Cumulative hectares and annual percent forest loss in the DRC from 2001–2021 (Hansen et al. 2013^30^ via global forest watch); (**b**) map of forest loss location (Hansen/UMD/Google/USGS/NASA) with gradient representing loss years from 2001 (yellow) to 2022 (red) alongside extent of primary humid tropical forest in 2001 (Turubanova et al. 2018^44^; Basemap: Bing); black dot represents location of (**c**) small scale agricultural expansion along the border of humid tropical forest and savanna. Maps developed using QGIS 3.28 (https://www.qgis.org/en/site/forusers/download.html) with post-processing in Adobe Illustrator 26.2.1 (https://www.adobe.com/products/illustrator.html).

Deforestation across intact tropical forests has been increasing in recent decades, with a total of 12% of forests lost between 2000 and 2020 (0.6%/year^30^). The DRC is considered an HFLD country—home to the majority of the remaining Congo Basin humid tropical rainforest^31^—with a rate of deforestation averaging 0.4% over the same period. However, this rate has more than doubled in the most recent decade (0.56%: 2011–2021) compared to the previous (0.25%: 2001–2010) (Fig. 7)^32^—with a total of 17.2 million hectares lost since 2001. The vast majority of this deforestation is a result of small-scale and/or subsistence agriculture^33,34^; particularly maize and cassava^35^ as opposed to commercial harvesting^36^. The expansion of forest loss is driven by high population growth and limited alternative livelihood opportunities^33,37^. While the rate of deforestation is relatively low, given the expansive area of forest in the DRC (147 million hectares^38^) and the high carbon stock therein^39–41^, recent forest loss has resulted in high greenhouse gas emissions^42^ as reflected in the country’s FREL (Fig. 1a). One defense against deforestation in the DRC and other countries is REDD+ , yet skepticism about baselines used in REDD+ projects threatens to stall the flow of finance to communities at the frontlines of deforestation.

There has been debate and concern about the future of baseline setting in the voluntary carbon market recently for multiple reasons. First, project developers and the market for carbon credits in general have received sharp criticism in the popular media; baselines and crediting levels have been the crux of these critiques. Second, accusations that carbon credits represent “hot air” have also burgeoned as buyers of credits have begun making claims around carbon neutrality and other environmental outcomes. Finally, as national governments continue to play a stronger role in regulating and coordinating REDD+ activities within their borders, existing baseline methodologies are being scrutinized in terms of how they align with national climate policies and ambitions. By presenting the BAAR method here, we aim to contribute towards increasing transparency around baseline setting in the voluntary carbon market and provide a new, efficient approach to establishing national risk maps which may be directly applied by national governments or carbon standards.

Such transparency not only fosters academic scrutiny and private-sector collaboration but also minimizes the likelihood of data manipulation (i.e. ‘gaming’). This necessitates uncertainty thresholding and a concerted effort by market actors to align with governments on maintaining relevance and quality of their national FRELs. Such an integrated public–private approach could further contribute to the credibility and clarity of a national emissions reduction program.

We also encourage transparency regarding the choices made in the allocation process. For example, reference level allocation can be a single or multi-stage process. In the single-stage case, a FREL is allocated directly to project activities. A multi-stage process involves distributing a FREL to a lower-level “subnational” administrative unit (e.g., province or territory), and the subnational FREL is then allocated to project activities. In countries with significant variation in the spatial distribution of deforestation, multi-stage allocation may better capture surrounding risk that is not inside, or adjacent to, site-scale activities. In Colombia, for example, a country that features a distinct deforestation frontier, distributing the national FREL in a single stage could result in underestimated baselines for projects in high deforestation departments that happen to be further from the frontier. Multi-stage allocation may also be appropriate in national contexts with federal systems where governance of forests is decentralized. A single-stage allocation is simpler to implement, and appropriate for contexts where high risk based on historical deforestation is more uniformly distributed nationally regardless of land tenure or administrative boundaries, as is the case for the DRC.

Another situation that may necessitate the adjustment of reference level allocation is when a jurisdiction exhibits wide variation in the spatial distribution of carbon stocks. In that case, using a pure risk-based approach without considering local carbon stock variability may yield project baseline allocations that under/over-estimate GHG emissions. We recommend in this case that a jurisdiction establish standards and a process for creating baseline adjustments. For example, a project reference level could be adjusted upwards to reflect the carbon stocks in the project area. If these adjustments are made transparently and accounted for within a jurisdiction’s total FREL, no double counting will occur and project developers will be incentivized to establish projects in areas with strong potential for GHG emission avoidance (high deforestation risk and high carbon stocks). In the case of the DRC, the mean carbon stock of the dense humid forest types (Table 1) are significantly higher than that of dry/open and secondary forests—thus, the weighting of FREL risk allocation by relative carbon stock was significant in producing accurate ER estimations. As a result of carbon stock weighting, the FREL allocation (tCO_2_e/year) was increased in 30 of the 34 tested projects—all located within dense humid forests. Circumstantial adjustments might also be considered in areas of planned deforestation—which require an entirely different method for baseline calculation—and for projects with pre-existing legal commitments from governments. Planned or adjusted FRELs can be removed from the allocation, and the allocation can then be re-run. Further adjustments could be made to the BAAR modeling results in the form of a discount applied to the estimated emissions reductions on a project-level. This discount could be based on uncertainties of the national FREL (carbon stock and activity data) as well as risk of reversals; however, this decision ultimately resides with the underlying jurisdictional REDD+ standard.

A final policy priority to consider is revenue from carbon credits. While most market concerns around inflated baselines focus on the reference levels calculated by project developers, one might imagine similar concerns being raised about how governments allocate a national FREL. However, if governments wish to maximize their own revenue share from REDD+ projects, experience from the petroleum sector suggests that there are far simpler policy tools at their disposal to do so, such as taxes, royalties, and operating fees^43^. Furthermore, as suggested by Fig. 5 above, it is difficult to predict the effect of varying BAAR parameters on project-level allocations, making it extremely difficult for any actor to “game” this method in a way that would undermine the integrity of the underlying GHG reductions.

## Conclusions

The BAAR deforestation prediction maps and allocation model presented here represent the underpinning of a jurisdictional REDD+ framework, providing open source data and code to ensure transparency and reduce potential favoritism, as a basis for a credible and trusted novel emissions reductions program. In this approach, the most significant spatial driver of future deforestation is past deforestation, applied to remaining forest cover using a dynamic, vector-based (non-uniform) approach. BAAR balances fitness for purpose with scientific rigor, allowing for simple nested project baseline calculation, resulting in distribution of incentive to areas determined to be at high risk of near-term deforestation.

We anticipate both GHG emission reduction programs and governments will adopt the BAAR method for determining nested REDD+ project baselines. This approach can be enhanced with supplementary risk variables to refine deforestation predictions within those jurisdictions. The next phase involves applying the BAAR methodology to additional key jurisdictions (see Fig. 1), facilitating the expansion of REDD+ initiatives in countries facing significant deforestation risks.

### Supplementary Information

Supplementary Information.

## Data Availability

Data available upon request.
