# Supplementary material for: A novel deforestation risk and baseline allocation model for the next generation of nested REDD+ projects
Source: Sci Rep. 2024 Jul 2;14:15138. doi: 10.1038/s41598-024-65141-x (PMC11219893; doi:10.1038/s41598-024-65141-x)
Supplement: Supplementary file 1 — Supplementary Information. [file 41598_2024_65141_MOESM1_ESM.docx]

**Supplementary Information**

**Forest reference level (FREL) for the Democratic Republic of the Congo (DRC)**

DRC’s FREL was submitted to the UNFCCC in 2018, and the report of the technical assessment was published by the UNFCCC in December of 2018. The modified FREL submission for the period 2015-2019 was an average of 1,078,235,017 t CO_2_ eq/year, constructed using historical data from 2000-2014. The FREL includes gross emissions from deforestation, and does not include emissions from degradation nor removals from regrowth in the deforested areas. LANDSAT imagery was used to calculate activity data. The FREL includes above-ground and below-ground biomass pools, and is limited to CO_2_ emissions.


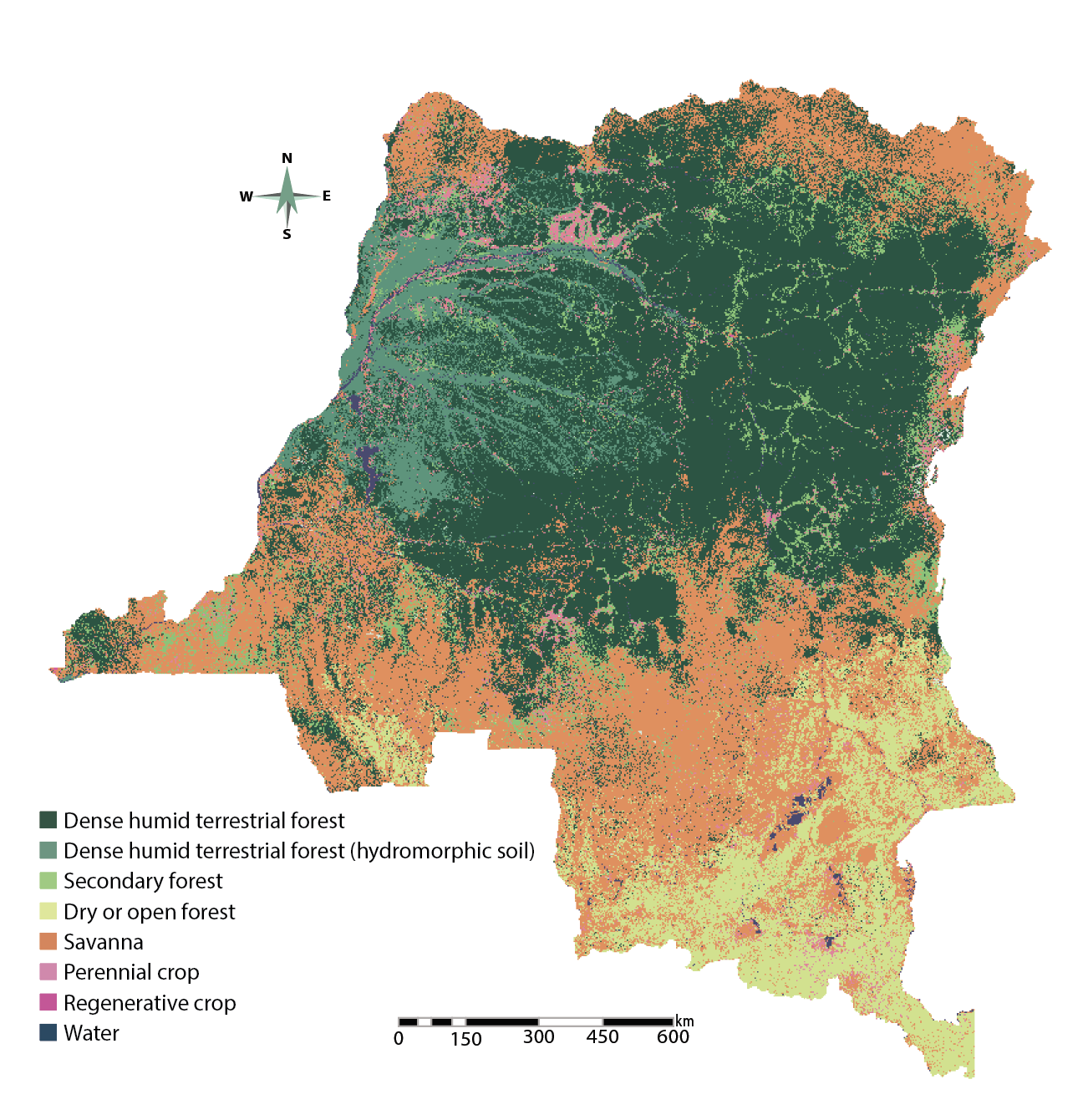


*Supplementary Figure 1: Forest cover benchmark map (year = 2000) used as a basis of the DRC FREL (MECNT, 2018). Map developed using QGIS 3.28 (https://www.qgis.org/en/site/forusers/download.html) with post-processing in Adobe Illustrator 26.2.1 (https://www.adobe.com/products/illustrator.html)*


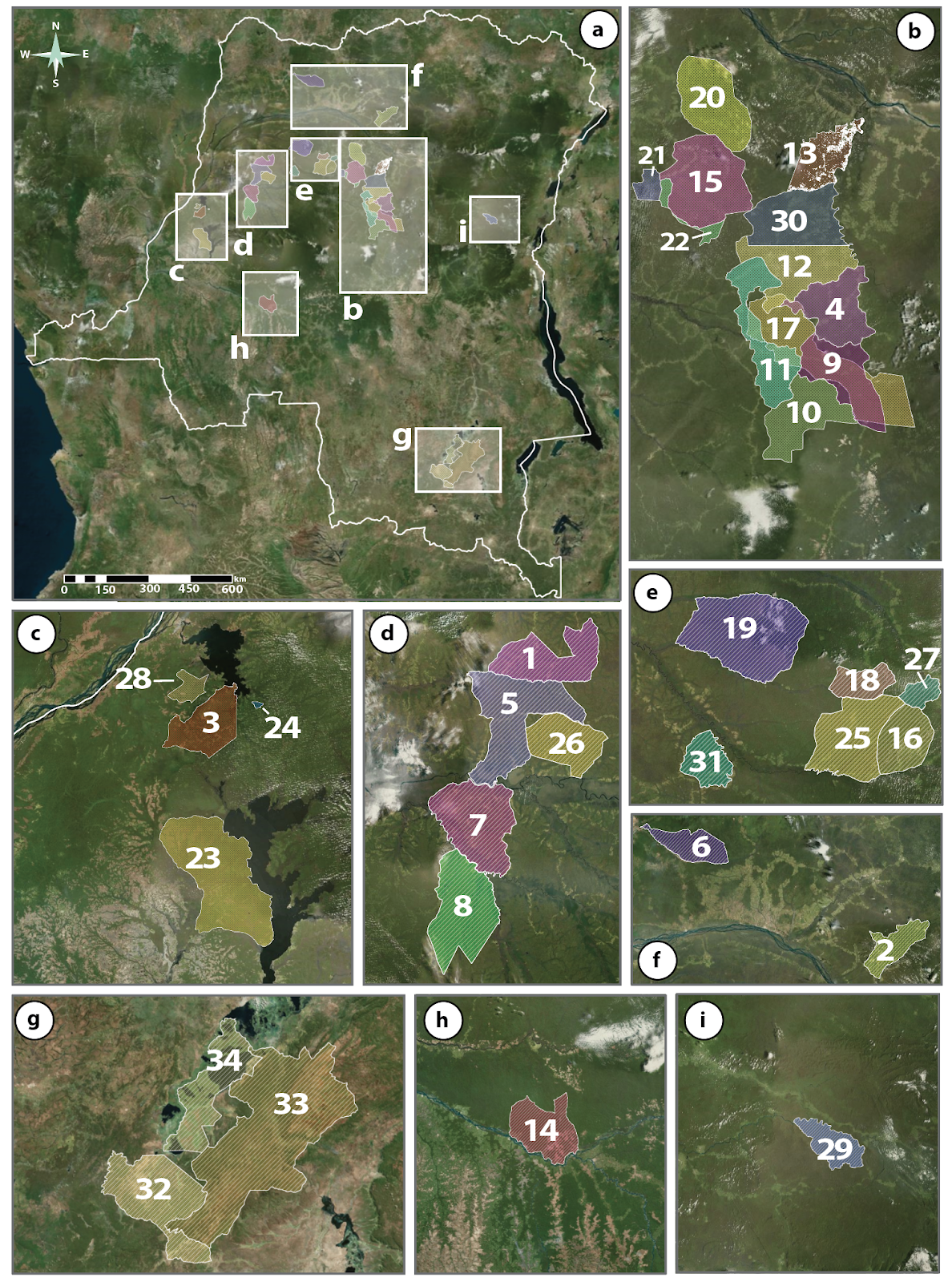


*Supplementary Figure 2: location of 34 selected REDD+ projects in various stages of development. Map developed using QGIS 3.28 (https://www.qgis.org/en/site/forusers/download.html) using a Bing aerial basemap (http://ecn.t3.tiles.virtualearth.net/tiles/a{q}.jpeg?g=1) with post-processing in Adobe Illustrator 26.2.1 (https://www.adobe.com/products/illustrator.html)*

*Supplementary Table 1: An overview of project accounting area and risk allocation results from the BAAR model run with risk multiple m=45.*

| ID | Project Accounting Area (ha) | Low Risk Area (ha) | High Risk area (ha) | Unweighted nested baseline (tCO2e/year) | Weighted nested baseline (tCO2e/year) |
| --- | --- | --- | --- | --- | --- |
| 1 | 161,138 | 141,491 | 19,408 | 636,312 | 741,162 |
| 2 | 274,410 | 269,479 | 4,862 | 406,333 | 480,804 |
| 3 | 93,741 | 68,047 | 24,979 | 698,615 | 806,724 |
| 4 | 216,417 | 157,331 | 58,278 | 1,628,446 | 1,928,278 |
| 5 | 271,484 | 250,085 | 21,024 | 791,448 | 919,836 |
| 6 | 270,528 | 211,634 | 58,894 | 1,701,182 | 1,995,423 |
| 7 | 228,941 | 211,893 | 16,714 | 642,994 | 741,780 |
| 8 | 236,726 | 209,850 | 26,555 | 887,794 | 1,026,916 |
| 9 | 274,186 | 243,455 | 29,950 | 1,008,442 | 1,189,586 |
| 10 | 289,196 | 267,802 | 21,099 | 812,012 | 952,577 |
| 11 | 273,415 | 228,141 | 44,201 | 1,349,898 | 1,577,935 |
| 12 | 227,853 | 145,183 | 81,585 | 2,200,487 | 2,605,160 |
| 13 | 165,043 | 139,044 | 25,862 | 795,700 | 942,216 |
| 14 | 208,542 | 186,080 | 21,136 | 726,712 | 860,172 |
| 15 | 406,400 | 374,695 | 31,073 | 1,175,094 | 1,382,773 |
| 16 | 117,339 | 99,390 | 17,754 | 550,393 | 650,813 |
| 17 | 506,680 | 457,164 | 48,280 | 1,693,911 | 1,997,982 |
| 18 | 55,058 | 43,674 | 11,243 | 328,224 | 388,270 |
| 19 | 352,959 | 325,809 | 26,528 | 1,009,467 | 1,186,057 |
| 20 | 301,488 | 266,260 | 34,370 | 1,143,428 | 1,352,708 |
| 21 | 43,366 | 35,915 | 7,288 | 220,771 | 260,047 |
| 22 | 43,147 | 40,973 | 2,115 | 96,295 | 112,908 |
| 23 | 212,999 | 102,486 | 109,538 | 2,856,878 | 3,321,239 |
| 24 | 1,414 | 936 | 467 | 12,702 | 14,826 |
| 25 | 211,304 | 175,945 | 34,987 | 1,063,607 | 1,255,707 |
| 26 | 140,511 | 130,313 | 10,136 | 391,849 | 457,490 |
| 27 | 29,436 | 23,367 | 6,032 | 176,021 | 208,075 |
| 28 | 24,132 | 18,002 | 6,034 | 170,414 | 196,816 |
| 29 | 102,999 | 91,748 | 10,997 | 372,767 | 441,407 |
| 30 | 225,071 | 139,877 | 84,065 | 2,257,123 | 2,672,328 |
| 31 | 226,939 | 71,386 | 11,021 | 351,894 | 412,276 |
| 32 | 491,605 | 104,518 | 107,252 | 2,801,674 | 1,087,768 |
| 33 | 31,655 | 349,223 | 134,250 | 3,737,357 | 1,456,520 |
| 34 | 31,655 | 22,139 | 8,654 | 240,524 | 93,385 |
